# Phytochemicals and Biological Properties of Azorean *Camellia sinensis* Black Tea Samples from Different Zones of Tea Plantation

**DOI:** 10.3390/plants14010103

**Published:** 2025-01-02

**Authors:** Lisete Sousa Paiva, Ana Paula Dias, Madalena Hintze Motta, José António Bettencourt Baptista

**Affiliations:** 1 Department of Science of Physics, Chemistry and Engineering of Faculty of Science and Technology and Institute of Agricultural and Environmental Research and Technology (IITAA), University of Azores, 9500-321 Ponta Delgada, Portugal; jose.ab.baptista@uac.pt; 2 Gorreana Tea Plantation, Gorreana, 9625-304 Maia, Portugal; ana-paula-dias-14@hotmail.com (A.P.D.); gorreanazores@gmail.com (M.H.M.)

**Keywords:** antioxidant properties, catechins and theaflavins content, phenolics, flavonoids and tannins content, tea health benefits

## Abstract

*Camellia sinensis* tea has received considerable attention due to its beneficial effects on health, particularly due to its antioxidant properties that are affected by several factors, which have a high influence on the final quality of black tea. The objective of this study was to investigate the biological properties of Azorean *C. sinensis* black tea from five different zones of tea plantation in order to select specific areas to cultivate tea rich in targeted compounds beneficial to human health. The free radical scavenging activity (FRSA), ferric reducing antioxidant power (FRAP), ferrous ion chelating (FIC) activities, total phenolic content (TPC), total flavonoid content (TFC), and tannins were determined by colorimetric methods, and catechin and theaflavin contents were analyzed by high-pressure liquid chromatography. The results indicated that samples from Zone E (341 m above the sea level) presented higher values of FRSA (EC_50_ = 7.22 µg/mL), FRAP (EC_50_ = 9.06 µg/mL), and FIC activities (79.83%) and higher values of total phenolics (264.76 mg GAE/g DE) and almost all catechins. For TFC, the values were very similar between zones, and for theaflavins content, Zone A showed the best levels, followed by Zone E. In general, these results clearly highlight that altitude plays a significant role in enhancing certain compounds of tea, thereby influencing its quality.

## 1. Introduction

Tea from *Camellia sinensis* (L.) Kuntze, belonging to the family Theaceae, is one of the most consumed aromatic beverages around the world and it is well known that *C. sinensis* teas have many important physiological properties and health benefits. The health benefits of tea consumption have been recognized for thousands of years around the world, especially for their polyphenols that are used in medicine, nutraceuticals, cosmetics, and food because of their antioxidant, anti-carcinogenic, antibacterial, anti-inflammatory, and anti-apoptotic properties [1,2].

Depending on the degree of fermentation, tea can be divided into three types, unfermented (green tea), semi-fermented (oolong tea), and fully fermented (black tea), that differ in terms of the manufacturing and chemical composition [3,4].

Black tea is the most popular tea produced worldwide, representing 70–80% of all tea consumption, followed by green tea (20%) and oolong tea (2%) [5].

The manufacturing process of black tea mainly includes withering, rolling, fermentation, and drying of the fresh leaves of *C. sinensis*, where withering and fermentation are critical steps for aroma and flavor of black tea development. During withering, cell sap of plucked shoots becomes concentrated and the leaves turn flaccid along with the moisture loss [6]. In black tea, the fermentation stage is a critical processing step where most of the polyphenols, such as catechins, are oxidized by oxidative enzymes, such as polyphenol oxidase (PPO) and peroxidase (PO), to form quinones which are then oxidized and condensed to form theaflavins (TFs), thearubigins (TRs), theabrownins, and other polymerization products [7,8]. These tea polyphenols received much attention because they have a broad spectrum of biological functions, such as antioxidant, anti-inflammatory, anti-tumor, anti-viral, antibacterial, and cardiovascular protection activities [9].

Polyphenols, which include catechins, theaflavins, tannins, and flavonoids, are the major bioactive ingredients present in tea liquor and are recognized as strong antioxidants. The radical scavenging activity, reducing power, and metal-chelating activity change according to their structural features. Tea polyphenols consist of many phenolic structural units that have different redox potentials [10].

Flavanols are the major polyphenolic compounds [11] found in fresh tea leaves and include flavan-3-ols (catechins), which comprise (−)-gallocatechins (GC), (−)-epicatechin (EC), (−)-epigallocatechin (EGC), (−)-epicatechin gallate (ECG), (−)-epigallocatechin gallate (EGCG), and (−)-gallocatechin gallate (GCG). During the manufacturing process of black tea, the catechins present in fresh tea leaves are oxidized both enzymatically and nonenzymatically to provide two main pigments: theaflavins and thearubigins, which are responsible for the color, taste, flavor, and aroma of the black tea brew. The four major theaflavins in black tea are theaflavin, theaflavin 3-gallate, theaflavin 3′-gallate, and theaflavin 3,3′-digallate, which are formed by bindings of EC and EGC, EC and EGCG, ECG and EGC, and ECG and EGCG, respectively [4,12].

The phytochemical composition of tea leaves is affected by several factors such as climatic conditions, varieties, location, brewing techniques, and processing conditions. The quality of black tea depends on the season of collection, altitude, plucking method, time and temperature of withering, fermentation, time and temperature of drying process, and also on storage conditions, which have a high influence on the final quality of black tea [13].

All plants produce phytochemical compounds, especially antioxidants that have pharmacological/therapeutical activities that can be used for applications in modern medicine [14]. These antioxidant compounds, especially from natural sources, are crucial for improving health and nutrition research [15]. Besides green, oolong, and black teas, a special type of tea called herbal tea has gained attention, and some of herbal tea’s benefits can be helpful when mixed with tea from *C. sinensis* [16]. According to Kaczyński et al. [17], tea and herbal tea can be contaminated by pesticides used in their production, but in the Gorreana Tea Plantation, all of the tea is produced free of pesticides and fungicides, because in the Azorean Islands, there are no tea pests, which gives greater importance to tea produced in the Azores.

This study aims to evaluate and compare the phytochemical and biological properties of Azorean *C. sinensis* black tea samples from different zones of tea plantation, in order to strategically select specific areas rich in target compounds that are beneficial to human health.

## 2. Results and Discussion

### 2.1. Yield

The yield percentages are described in Table 1 and vary between 27.60 and 31.30% for Zone D and E, respectively. Zone B (31.20%) presented similar and non-significant results related to Zone E.

### 2.2. In Vitro Antioxidant Activity

The in vitro scavenging and/or antioxidant activity of a certain substance is generally evaluated using chemical-based assays. In this study, the assays used were the DPPH free radical scavenging activity (FRSA), the ferric reducing antioxidant power (FRAP), and the ferrous ion chelating (FIC) activity.

For the antioxidant properties based on DPPH-FRSA, the results observed in Table 1 presented a higher ability to scavenge DPPH radicals in the sample from Zone E (EC_50_ = 7.22 µg/mL), followed by Zone A (EC_50_ = 10.36 µg/mL) and B (EC_50_ = 10.45 µg/mL). Furthermore, samples from Zone C and D presented lower abilities to scavenge DPPH radicals, with EC_50_ values of 12.57 and 15.01 µg/mL, respectively. Our black tea samples presented better results than other samples reported by some authors [18,19] that presentedEC_50_ values of 51.88 µg/mL and 497 µg/mL for black tea samples. However, Deo et al. [20] presented results that are comparable to our study (18.45–21.29 µg/mL).

Based on the FRAP results that are showed in Table 1, the same pattern was observed with the highest antioxidant activity for samples from Zone E (EC_50_ = 9.06 µg/mL), followed by Zone B and A (EC_50_ = 11.00 and 11.15 µg/mL, respectively). Zone C and D presented the lowest antioxidant activity with values of 14.70 and 15.34 µg/mL, respectively. Tong et al. [21] observed an EC_50_ value of 194 µg/mL, representing a lower antioxidant activity as compared to our results.

For FIC activity, the results are presented in Table 1, and the highest values were obtained for samples from Zone E (79.83%), followed by Zone C (69.80%) and Zone D (68.13%). The lowest values were observed in samples from Zone B (58.85%). The iron chelating activity of tea sample extracts demonstrated in this study could be related to their amount of total phenolic and flavonoid contents.

In general, the sample from Zone E presented higher antioxidant properties in relation to other zones in this study, mainly due to the higher altitude of this zone (341 m above sea level), which influences the metabolism of the tea plant and consequently the antioxidant activity.

According to Lee et al. [22], tea extracts enriched with phenolic compounds showed strong antioxidant activity and that could be attributed to the high contents of EGCG, ECG, and EGC. However, the antioxidant activity of tea samples not only depends on the levels of antioxidant compounds but also on their synergistic and/or antagonistic effects.

### 2.3. Total Phenolic, Flavonoid, and Tannin Contents

**Figure 1:**
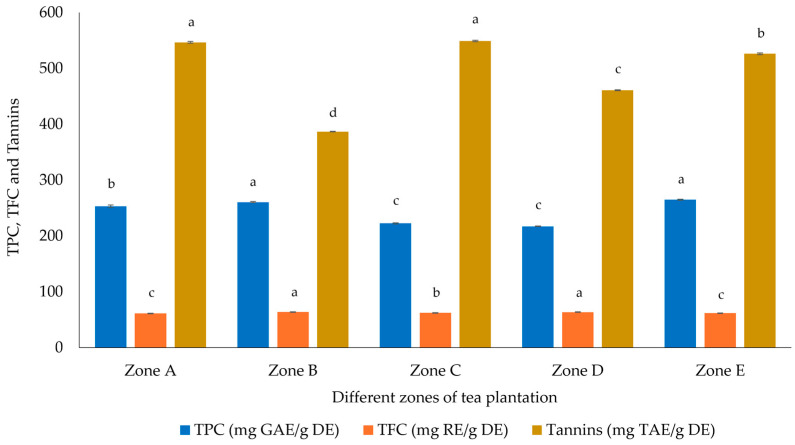
Total phenolic, flavonoid, and tannin content in Azorean *Camellia sinensis* black tea from different plantation zones GAE, gallic acid equivalents; RE, rutin equivalents; TAE, tannic acid equivalents; DE, dry extract. Different superscript letters indicate that values are significantly different (*p* < 0.05) between zones for the same column color.

The TPC results observed in Figure 1 and expressed in milligrams of GAE/g DE, showed that the highest value was observed for samples from Zone E (264.76 mg GAE/g DE), followed by Zone B (260.48 mg GAE/g DE) and the lowest value in Zone D (217.09 mg GAE/g DE). In addition to antioxidant activity, Zone E presented higher phenolic contents and higher epicatechin derivatives (sum of EC, EGC, EGCG, and ECG), and the same results were also observed for total catechins, demonstrating that polyphenols were the compounds that contributed the most to the antioxidant activities.

The results from Rohadi et al. [23] and Rahman et al. [24] are comparable to our results (256.7 mg and 224.69–254.62 mg GAE/g DE, respectively).

However, Atalay and Erge [18] and Chan et al. [25] presented significantly lower TPC values (58.20–61.94 and 60.61–84.94 mg GAE/g, respectively). Chang et al. [26] also observed lower values as compared to our results (approximately between 150 and 200 mg GAE/g). Although, Deo et al. [20] also reported comparable and higher values in relation to our results (191.62–301.94 mg GAE/g).

Regarding the TFC results shown in Figure 1 and expressed in milligrams of RE/g DE, the flavonoid content is similar between samples, with higher values in samples from Zone B and D with 63.79 and 63.78 mg RE/g DE, respectively, and the lowest value in the sample from Zone A, with 61.25 mg RE/g DE. These values are related to the results described by Rahman et al. [24], are higher than the results reported by Atalay and Erge [18], and are lower than the results presented by Yadav et al. [19].

The tannin content also illustrated in Figure 1, and expressed in milligrams of TAE/g DE, showed that samples from Zone A, C, and E presented higher levels, with values of 546.36, 548.97, and 525.90 mg TAE/g DE, respectively, and lower levels were present in samples from Zone B, with 386.79 mg TAE/g DE. Yadav et al. [19] showed lower values for tannins in black tea samples (approximately 85 mg GAE/g DE). This difference may be related to differences in terms of the extraction/analysis methodologies used, as well as genetic differences, geographic location, processing, and storage conditions.

### 2.4. Phenolic Compounds and Caffeine (CAF) Content

The data presented in Table 2 reveals the phenolic compounds (catechins) in the tea extracts, with a significant difference among tea samples from different tea plantation zones in terms of major catechins, epicatechin derivatives (ECDs), esterified (EGCG + ECG + GCG) and non-esterified catechins (C + EC + EGC + GC) and caffeine (CAF) contents. The analytical conditions of the HPLC method developed for the catechins’ determination were as follows: *R*^2^ range between 0.9965 and 0.9995 that indicates good linearity. The results also show the LOD that ranged from 0.01 to 0.20, and the LOQ that ranged from 0.05 to 0.55 mg/L, in the experimental condition used (see Table S1 in Supplementary Materials).

The results showed that the total catechins vary between 7.21 and 48.46 mg/g for a dry extract (DE), with the highest value in the sample from Zone E and the lowest value in the sample from Zone D, and the same pattern was observed for ECDs and esterified and non-esterified catechins, with values between 5.36 and 42.99 mg/g DE, 4.18 and 33.80 mg/g DE, and 2.49 and 13.68 mg/g DE, respectively. The major catechins in all tea samples were EGCG, ECG, and EC, with the highest values from the sample from Zone E and the lowest values for the sample from Zone D. For EGCG, the values vary between 1.61 and 16.57 mg/g DE. For ECG, the values varied between 2.19 and 16.43 mg/g DE and for EC between 1.13 and 6.52 mg/g DE. The GC, EGC, C, and GCG were the catechins that presented lower values.

Regarding CAF content, the values ranged from 16.66 to 55.22 mg/g DE with the highest value in the sample from Zone E and the lowest in the sample from Zone B. The samples from Zones A, C, and D presented no significant results, with values of 22.76, 22.25, and 29.75, mg/g DE, respectively.

Some authors presented different results for black tea samples. Koch et al. [27] observed that the major catechin was EGC and the least abundant was EC, and Leung et al. [28] described that ECG was the major catechin. Wang et al. [29] reported that ECG was also the major catechin in their samples with values between 0.16 and 25.66 mg/g, between 1.40 and 15.89 mg/g for EGCG, between 0.21 and 5.85 mg/g for EGC, and between 0.07 and 5.19 mg/g for EC.

Atalay and Erge [18] reported lower values for the catechins with respect to our results, and that may be related with differences in the analytical methodologies and biotic and/or abiotic factors. However, for CAF content, the results from these authors can be compared to our samples from Zone A, C, and D. Wang et al. [29] reported values between 14.81 and 36.22 mg/g that are comparable to our samples from Zones A, B, C, and D.

The higher values observed in Zone E, for almost all catechins, as already discussed, can be explained by the higher altitude (341 m above sea level) of the tea plantation and by the different climatic conditions. Lee et al. [30] also described that the combined EGCG and ECG content has been considered a key factor that affects the antioxidant activity of tea extracts, which can explain the higher antioxidant activity of the samples from Zone E with higher levels of EGCG and ECG.

It is important to point out that some conditions may influence the tea catechin levels, and these may be related to genetic variation, geographical location, growth altitude, different agricultural practices, soil types and fertility, sunlight intensity, temperatures, rainfall distribution, and water stress [31,32]. These conditions affect the tea and may be explained by the higher values of catechins in Azorean black tea samples.

### 2.5. Theaflavins Content

Table 2 also shows the differences in black tea samples for theaflavin contents in different zones of the tea plantation, and the results revealed that for total theaflavins, the sample from Zone A presented higher values, followed by the sample from Zone E, with 36.73 and 32.87 mg/g DW, respectively. For the individual theaflavins, the same pattern was observed. Related to TFs, the samples presented values that range from 6.13 to 8.32 mg/g DW; for TF-3-G, the results range from 6.60 to 9.92 mg/g DW; for TF-3’-G, they range from 6.06 to 9.73 mg/g DW; and for TF-3,3’-DG, they range from 5.58 to 9.04 mg/g DW. The higher levels of theaflavins observed in Zone A may be attributed to the relative youth of the plantation compared to the other studied zones. The second-highest levels were found in the sample from Zone E, which corresponds to a plantation located at a higher altitude.

Some authors [5,33,34] presented lower values for all theaflavins, while Ramdani et al. [35] presented comparable values for TF-3,3′-DG (6.98 mg/g) but lower results for the total theaflavins (16.7 mg/g) in comparison to the values obtained in our study (24.37–36.73 mg/g DW). Wang et al. [29] also reported lower values (5.27 mg/g DW) of theaflavins compared to our study, with the exception of TF-3,3’-DG.

The analytical conditions of the HPLC method developed for the catechins’ determination were as follows: the *R*^2^ ranged between 0.9993 and 0.9999, indicating good linearity. The results also show a LOD that ranged from 0.01 to 0.35, and the LOQ ranged from 0.33 to 1.25 mg/L, in the experimental condition used (see Table S2 in Supplementary Materials).

It is well known that theaflavin contents are related to the taste and quality of black tea [36], and according to some authors [37,38], theaflavins, especially TF-3,3′-DG, also presented anti-viral properties. All samples have an excellent content of theaflavins and TF-3,3′-DG, representing a potential inhibitor of SARS-CoV-2 and, consequently, important benefits for human health.

### 2.6. Pearson’s Correlation

**Figure 2:**
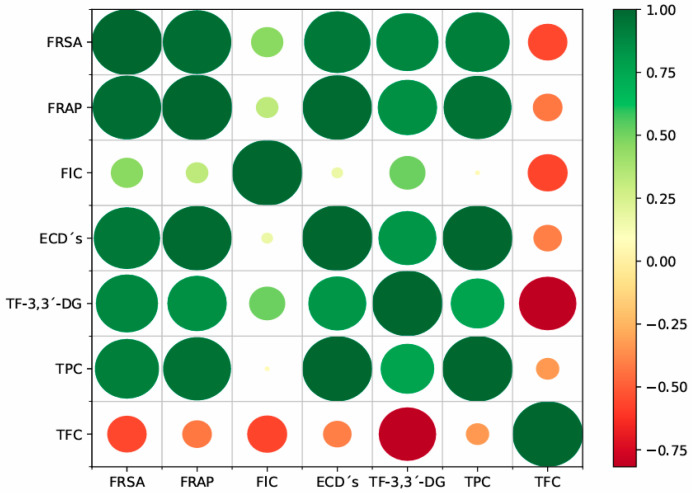
Correlation matrix of the studied parameters in Azorean *Camellia sinensis* black tea (Pearson’s correlation coefficients). FRSA, free radical scavenging activity; FIC, ferrous ion chelating; ECDs (epicatechin derivatives), sum of EGC, EC, EGCG, and ECG (epigallocatechin, epicatechin, epigallocatechin-gallate, and epicatechin-gallate); TF-3,3’-DG, theaflavin-3,3′-*O*-digallate; TPC, total phenolic content; TFC, total flavonoid content.

Pearson’s correlations are represented in Figure 2 and are used to assess the relationship between biological activities, such as antioxidants (FRSA, FRAP, and FIC), catechin derivatives, TF-3,3′-DG, and total phenolic and flavonoid contents. In general, the results presented a positive correlation. FRSA values were strongly correlated with FRAP, ECDs, TPC, and TF-3,3′-DG (*r* = 0.973, 0.942, 0.911, and 0.884, respectively). FRAP was also strongly correlated with ECDs, TPC, and TF-3,3′-DG (*r* = 0.984, 0.964, and 0.845, respectively). ECDs also presented a strong correlation with TPC and TF-3,3′-DG (*r* = 0.994 and 0.825, respectively). TF-3,3′-DG shows a strong correlation with TPC and FIC (*r* = 0.763 and 0.514, respectively). Some negative strong correlations were observed between TF-3,3′-DG and TFC (*r* = −0.817), between FIC and TFC (*r* = −0.569), and between FRSA and TFC (*r* = −0.561). Positive and moderate correlations were shown between FRSA and FIC (*r* = 0.460) and between FRAP and FIC (*r* = 0.320). Only two weak correlations were observed between FIC and ECDs (*r* = 0.166) and between FIC and TPC (*r* = 0.068).

## 3. Conclusions

These results clearly highlighted differences in the bioactivity and quality of Azorean black tea according to different zones of the tea plantation. The tea sample from Zone E (341 m above the sea level) was the sample that presented higher values of antioxidant properties, namely for FRSA, FRAP, and FIC activities, and shows higher values of total phenolics and almost all catechins (ECDs, esterified and non-esterified). These results indicate that these methods are also strongly correlated.

For theaflavins, the best results were from Zone A, followed by Zone E, and for TFC, the values were very similar between zones, indicating that different altitude zones do not interfere with total flavonoid content. For tannin content, the best zone was Zone C, followed by Zone A and E.

In general, these results indicate that altitude plays a significant role in enhancing certain components of tea, thereby influencing its quality. By understanding the optimal plantation zones for producing beneficial compounds to human health, we can strategically select specific areas to cultivate tea rich in these targeted compounds.

## 4. Materials and Methods

### 4.1. Chemicals and Reagents

Catechins, namely (−)-gallocatechin (GC, 98%—G6657), (+)-catechin (C, 98%—C1251), (−)-epicatechin (EC, 98%—E4018), (−)-epigallocatechin (EGC, 98%—E3768), (−)-epigallocatechin-3-gallate (EGCG, 95%—E4143), and (−)-epicatechin-3-gallate (ECG, 98%—E3893), as well as (−)-gallocatechin 3-gallate (GCG, 98%—G6782), caffeine (CAF, 99%—C0750), gallic acid (98%—G7384), theaflavin (TF), theaflavin-3-O-gallate (TF-3 G), theaflavin-3′-O-gallate (TF-3′-G), theaflavin-3,3′-O-digallate (TF-3,3′-DG), rutin, 2,2-diphenyl-1-picrylhydrazyl (DPPH), ethylenediaminetetraacetic disodium salt (EDTA), potassium ferricyanide (K_3_FeCN_6_), iron (II) chloride (FeCl_2_), iron (III) chloride (FeCl_3_), aluminum chloride (AlCl_3_), ferrozine, trichloroacetic acid (TCA), and Folin–Ciocalteu reagent (FCR) were all obtained from Sigma-Aldrich (St. Louis, MO, USA). Sodium chloride (NaCl), sodium phosphate (NaH_2_PO_4_), sodium carbonate (Na_2_CO_3_), potassium acetate (KCH_3_CO_2_), and formic acid were obtained from E. Merck (Darmstadt, Hessen, Germany). HPLC-grade methanol (MeOH) and acetonitrile (ACN) were purchased from Fluka Chemika (Steinheim, Switzerland). Chloroform and ethyl acetate, HPLC-grade, were obtained from Riedel-de Häen (Aktiengesellschaft, Seelze, Germany). Ultrapure glass-distilled water that was deionized with a Millipore Milli-Q purification system (Millipore, Bedford, MA, USA) was used throughout all the experiments.

### 4.2. Tea Samples and Tea Extract Preparation

Tea leaves from Azorean *Camellia sinensis* (L.) Kuntze var. *sinensis* were collected from five different zones with different altitudes (Zone A: 205 m; Zone B: 212 m; Zone C: 235 m; Zone D: 242 m; and Zone E: 341 m above the sea level) in the Gorreana Tea Plantation (São Miguel Island, Azores, Portugal—37°49′06″ N 25°24′08″ W). The leaves were first separated, by the apical buds and the two youngest leaves, and were cultivated in volcanic soil, with an average pH = 5.6 (range of 4.1–6.3), rich in the basic elements: N = 0.17 g/100 g, P = 58 mg/Kg, K = 0.49 meq/100g, and Mg = 0.50 meq/100 g, expressed per weight of dried soil (average data obtained from the Gorreana Tea Plantation). The tea samples were prepared under the following conditions: tea leaves freshly plucked were indoor-withered at 25–30 °C for 15 h, oxidized for 3 h, and dried in a heating chamber at 70 °C with a rotating fan to keep the heat evenly distributed. Then, tea leaves were grounded in a mortar to a particle size of 20–30 µm and refrigerated under an atmosphere of N_2_ until further experimental steps.

*C. sinensis* black tea samples were prepared in the following conditions: 1 g of dried powder material was extracted with 20 mL of distilled water and heated at 70 °C in a water bath with mild stirring (250 rpm) for 15 min to avoid the degradation of compounds that can occur at temperatures higher than 70 °C. The extraction process was repeated three times, and the combined extract was filtered under vacuum through a cellulose acetate membrane (porosity of 0.45 µm) to remove particulate matter. The extract was dried on a rotary evaporator and lyophilized for further analysis.

### 4.3. Determination of Yield

The yield percentage was determined to analyze the effect of the solvent system on the extraction and was calculated using the following equation: Yield (%) = 100 × (A − B)/W, where A = weight of flask containing extract after evaporation, B = weight of dry empty flask, and W = weight of dry sample.

### 4.4. Determination of the In Vitro Antioxidant Activity

Several methods are used to investigate the antioxidant property of plant extracts. To evaluate the antioxidant properties of bioactive natural compounds, the samples under study were evaluated using different in vitro antioxidant assays that are the three major antioxidant assays, such as scavenging various types of free radicals or reactive oxygen species (DPPH-FRSA), assessing reducing power (FRAP), and examining metal chelation (FIC).

#### 4.4.1. Determination of DPPH-Free Radical Scavenging Activity (FRSA)

Free radical scavenging activity of *Camellia sinensis* extract was measured using 1,1-diphenyl-2-picrylhydrazyl (DPPH) according to the method of Tong et al. [21] with slight modifications [39]. In this test, a stable free radical (DPPH) is combined with the plant extract, and the amount of color change is measured based on the color of the reagent’s mixture from purple to bright yellow, and the intensity of this change was monitored spectrophotometrically. An aliquot of 250 µL of each extract sample was mixed with 500 µL of 100 µM DPPH solution. Ascorbic acid was used as the reference sample at the same concentration of the extracts, and a mixture without the sample, or ascorbic acid, was used as the control. The solution was incubated at room temperature in the dark for 30 min, and the Abs was measured at 517 nm. The FRSA was calculated as a percentage of DPPH-induced discoloration using the following equation: FRSA (%) = (1 − Abs_sample_/Abs_control_) × 100.

The EC_50_ value (μg/mL) represented the extract concentrations required to neutralize 50% of the DPPH and the value was obtained by interpolation from linear regression analysis. A lower EC_50_ value was indicative of higher antioxidant activity.

#### 4.4.2. Determination of Ferric Reducing Antioxidant Power (FRAP)

The FRAP assay acts by reducing the ferric ions to ferrous ions (Fe^3+^ complex to Fe^2+^) through antioxidants present in the samples, and a blue color is developed which is read colorimetrically. FRAP was determined according to the method of Oktay et al. [40] with some modifications [39]. An increase in the Abs values indicates an increased reducing power of the samples. An aliquot of 0.4 mL of each extract sample with 0.4 mL of 200 mM of phosphate buffer (pH = 6.6) and 0.4 mL of potassium ferricyanide (1%, *w*/*v*) was incubated for 20 min at 50 °C. After cooling down, 0.4 mL of TCA (10%, *w*/*v*) was added, and the mixture was centrifuged at 4000× *g* for 10 min. The upper layer (1 mL) was mixed with an equal amount of deionized water plus 0.2 mL of FeCl_3_ (0.1% *w*/*v*). Ascorbic acid was used as a standard sample. The results were expressed as an EC_50_ value (µg/mL), which is the concentration at which the Abs was 0.5 for reducing power and were obtained by interpolation from linear regression analysis of concentration versus Abs at 700 nm against a blank. A lower EC_50_ value indicates higher antioxidant activity.

#### 4.4.3. Determination of Ferrous Ion Chelating (FIC) Activity

The FIC activity assay acts as metal chelating capacity and is claimed to be the most important mechanism which underpins antioxidant activity [41]. FIC activity was estimated according to the method of Wang et al. [41] with some modifications [39]. The chelating ability of each extract, at various concentrations, was measured following the inhibition of the formation of the Fe^2+^–ferrozine complex, after the incubation of samples with iron. Briefly, an aliquot of 100 µL of each extract sample (5 mg/mL) was mixed with 370 µL of methanol plus 10 µL of 2 mM FeCl_2_. The reaction was initiated by the addition of 20 µL of 5 mM ferrozine, shaking the sample, and then leaving the sample standing for 10 min at room temperature. The mixture was measured spectrophotometrically at 562 nm. Methanol, instead of ferrozine solution, was used as a blank sample, which is required for error correction because of the unequal color of the sample solutions. Methanol, instead of a sample solution, was used as a control. Results were expressed as relative iron chelating activity compared with the unchelated (without ferrozine) Fe^2+^ reaction, and EDTA was used as the reference standard. A lower Abs was indicative of better FIC activity. The FIC activity was calculated as follows: FIC activity (%) = (A0 − (A1 − A2))/A0 × 100, where A0 was the Abs of the control, and A1 was the Abs of the sample or standard, while A2 was the Abs of the blank.

### 4.5. Determination of the Total Phenolic Content

Phenolic compounds can capture free radicals and metal chelators, working at the beginning of oxidation and the propagation process. 

Total phenolic content (TPC) was determined by using the Folin–Ciocalteu (FCR) colorimetric methodology through a technique described by Wang et al. [42], with some modifications [39]. An aliquot of 100 µL of each extract sample (2 mg/mL) was mixed with 1500 µL of distilled water and 100 µL of 2N FCR, homogenized in a vortex for 15 s, and allowed to sit in the dark for 3 min. Then, 300 µL of 10% Na_2_CO_3_ (*w*/*v*) was added and homogenized and incubated for 5 min at 50 °C for blue color development, and the absorbance (Abs) was measured at 760 nm. Gallic acid was used to produce a standard calibration curve at various concentrations, and the results were expressed in milligrams of gallic acid equivalents per gram of dried extract (mg GAE/g of DE).

### 4.6. Determination of the Total Flavonoid Content

The total flavonoid content of the crude extract was determined by the aluminum chloride colorimetric method [43] with slight modifications [39]. An aliquot of 100 µL of each extract sample (2 mg/mL) was mixed with 900 µL of distilled water, followed by the addition of 100 µL of 10% AlCl_3_ and 100 µL of 10% KCH_3_CO_2_. The mixture was homogenized in a vortex for 15 s and allowed to stand for 30 min at room temperature, followed by measuring the Abs at 415 nm. Rutin was used to produce a standard calibration curve at various concentrations, and the results were expressed as mg of rutin equivalents per gram of dried extract (mg RE/g of DE).

### 4.7. Determination of the Total Tannin Content

The total tannins content was determined by using Folin–Ciocalteu colorimetric methodology described by Haile and Kang [44] with some modifications. A 125 µL aliquot of a 5 mg/mL extract was combined with 4375 µL of distilled water and 250 µL of Folin–Ciocalteau phenol reagent. The mixture was then allowed to incubate at room temperature for 5 min. Afterwards, a volume of 500 µL of 35% Na_2_CO_3_ was added, and the solution was vigorously agitated and allowed to rest at room temperature for 20 min, and the absorbance was measured at 725 nm. A series of standardized solutions containing tannic acid were measured using a blank as a reference. The results were quantified in milligrams of tannic acid equivalents per gram of dry extract (mg TAE/g of DE).

### 4.8. Determination of Phenolic Compounds and Caffeine (CAF) Content

The extraction of phenolic compounds (crude catechins) and CAF was carried out by the method described by Baptista et al. [45] with slight modifications. An amount of 100 mg of tea extract was mixed with Milli-Q water until a final volume of 25 mL. Briefly, an aliquot of 10 mL was partitioned with an equal liquid volume of chloroform to remove pigments and other non-polar plant material. Then, the same volume of ethyl acetate was added to the aqueous solution. The extraction was repeated three times, and the three extracts were combined. Ethyl acetate was evaporated in a vacuum rotary evaporator, and the light-brown residue, called “crude catechins”, was dissolved until there was 500 µL of water and then subjected to high-performance liquid chromatography/photodiode array detection (RP HPLC/PDAD).

#### 4.8.1. RP-HPLC Analysis of Phenolic Compounds and Caffeine (CAF)

To quantify the individual phenolic compounds and caffeine content, the following method of Baptista et al. [45] was used and carried out by a RP-HPLC method (Waters, Milford, MA, USA) using a Phenomenex Synergi C12—4 µm MAX-RP 80Å (150 × 4.6 mm i.d.) column. The mobile phase was composed of mobile phase A with water–formic acid (99:1, *v*/*v*), while mobile phase B was acetonitrile–formic acid (99:1 *v*/*v*). The separation was achieved by a linear gradient in the following conditions: t = 0 min—4%B; t = 60 min—25%B; flow rate of 0.7 mL/min. The temperature of the column was maintained at 40 °C, and a Waters 486 tunable absorbance detector fixed at 280 nm was used. The quantitative determinations were performed using a Hewlett-Packard integrator model HP-3396 series II. An aliquot of 5 µL was subjected to HPLC. The chromatograms were recorded according to the retention times (RTs), and the quantitative analysis was achieved by the external standard method. The catechin sample concentration was limited to the range of linearity. Peak identity was assigned based on the RT following the comparison with the authentic standards. The results were expressed as milligrams per gram of sample on a dried extract (DE) basis. The multilevel working calibration curves at five different concentrations, plus the linear range in mg/L for the catechins, were determined, as well as the limit of detection (LOD) and limit of quantification (LOQ), which are defined as the amount of injected sample that give a signal-to-noise ratio of 3 and 10, respectively.

### 4.9. Sample Preparation for Theaflavin Determination

The extraction of theaflavins from different tea samples was performed following the method of Matsubara and Rodriguez-Amaya [46] with slight modifications [47]. Briefly, 200 mg of each dry tea sample was brewed with a 20 mL solution (8:2, methanol–water) for 30 min at 70 °C with mild stirring (250 rpm). The solution was filtrated through a 0.45 m (pore size) cellulose acetate membrane, and 10 mL of solution was concentrated in a rotary evaporator and reconstituted in 2 mL of methanol. An aliquot of 12.5 µL was injected into the HPLC device.

#### RP-HPLC Analysis of Theaflavins

The concentration of theaflavins in each tea extract samples was quantitatively assessed using HPLC (Agilent 1200) with an Omnisphere 3 µm (100 × 4.6 mm i.d.) column from Varian, under conditions of a two-solvent gradient: mobile phase A was composed of H_2_O–formic acid (99.9:0.1, *v*/*v*), while mobile phase B was methanol–formic acid (99.9:0.1, *v*/*v*). Chromatographic separation was achieved by a linear gradient, and the elution gradient consisted of the following parameters: t = 0 min—35% B; t = 35 min—35%B; t = 60 min—50% B; and a flow rate of 0.8 mL/min. The column, maintained at 40 °C, was attached to a 1260 Infinity II Quaternary Pump Liquid Chromatograph System, equipped with a PDAD fixed at 365 nm. The quantitative analyses were performed according to the external standard method using the Open Lab CDS VL Workstation software v2.4 from Agilent Technologies (Avondale, PA, USA). Peak identification was assigned by comparison with the authentic standards and further confirmed by superimposing the spectrum of each peak with the corresponding standard spectrum. The results were expressed as mg/g of the sample on a dry weight (DW) basis. The multilevel working calibration curves at five different concentrations, plus the linear range in mg/L for the theaflavins, were determined. The limit of detection (LOD) and limit of quantification (LOQ) were obtained using the same methodology described in Section 4.8.1.

### 4.10. Statistical Analysis

All the data were reported as the mean ± standard deviation (SD) of three replicates. A one-way analysis of variance test (ANOVA) was carried out to assess and indicate any significant differences between the mean values obtained from each sample. Significance was based on a confidence level of 95% (*p* < 0.05). The statistical analysis was performed using SPSS 20.0 (SPSS Inc., Chicago, IL, USA). Correlations between the tea quality and evaluated parameters were obtained using Pearson’s correlation coefficient (*r*) by Python 3.8 and Matplotlib 3.7.1.

## Figures and Tables

**Table 1:** Antioxidant activities (free radical scavenging activity—FRSA, ferric reducing antioxidant power—FRAP, and ferrous ion chelating—FIC) in Azorean *Camellia sinensis* black tea from different plantation zones ^1^.

	Yield (%)	FRSA(EC_50_, µg/mL)	FRAP(EC_50_, µg/mL)	FIC(%)
Zone A	30.20 ^b^	10.36 ± 0.04 ^c^	11.15 ± 0.13 ^c^	67.02 ± 1.82 ^c^
Zone B	31.20 ^a^	10.45 ± 0.06 ^c^	11.00 ± 0.35 ^c^	58.85 ± 0.68 ^d^
Zone C	29.00 ^c^	12.57 ± 0.35 ^d^	14.70 ± 0.23 ^d^	69.80 ± 1.80 ^c^
Zone D	27.60 ^d^	15.01 ± 0.33 ^e^	15.34 ± 0.25 ^e^	68.13 ± 1.44 ^c^
Zone E	31.30 ^a^	7.22 ± 0.23 ^b^	9.06 ± 0.11 ^b^	79.83 ± 0.51 ^b^
Ascorbic acid	-	2.11 ± 0.04 ^a^	3.91 ± 0.27 ^a^	-
EDTA	-	-	-	98.16 ± 0.15 ^a^

^1^ Values are mean ± SD (*n* = 3); Different superscript letters indicate that values are significantly different (*p* < 0.05) between zones; EC_50_, half-maximal effective concentration; EDTA, ethylenediamine–tetra acetic disodium salt. Different tea plantation zones are harvested at different altitudes above sea level (Zone A: 205 m; Zone B: 212 m; Zone C: 235 m; Zone D: 242 m; and Zone E: 341 m).

**Table 2:** Catechins and caffeine content (mg/g of the sample for a dry extract) and theaflavins content (mg/g of the sample for dry weight) in Azorean *Camellia sinensis* black tea from different plantation zones ^1^.

Phenolics Compounds and Caffeine	Zone A	Zone B	Zone C	Zone D	Zone E
GC	1.03 ± 0.01 ^ab^	0.79 ± 0.11 ^bc^	0.71 ± 0.01 ^cd^	0.42 ± 0.01 ^d^	1.33 ± 0.03 ^a^
EGC	1.60 ± 0.27 ^c^	2.36 ± 0.04 ^b^	1.15 ± 0.04 ^c^	0.43 ± 0.01 ^d^	3.47 ± 0.02 ^a^
C	1.19 ± 0.04 ^bc^	1.63 ± 0.05 ^b^	0.88 ± 0.04 ^cd^	0.51 ± 0.01 ^d^	2.36 ± 0.29 ^a^
EC	5.79 ± 0.48 ^b^	6.50 ± 1.39 ^a^	1.58 ± 0.10 ^c^	1.13 ± 0.09 ^c^	6.52 ± 0.30 ^a^
EGCG	12.95 ± 0.25 ^b^	11.50 ± 0.45 ^c^	2.88 ± 0.13 ^d^	1.61 ± 0.13 ^e^	16.57 ± 0.49 ^a^
GCG	1.37 ± 0.07 ^b^	2.84 ± 0.19 ^a^	0.63 ± 0.04 ^c^	0.38 ± 0.13 ^d^	0.80 ± 0.09 ^c^
ECG	12.80 ± 0.10 ^c^	14.34 ± 0.58 ^b^	3.00 ± 0.01 ^d^	2.19 ± 0.40 ^d^	16.43 ± 1.21 ^a^
GA	4.90 ± 0.10 ^c^	12.90 ± 0.32 ^a^	2.79 ± 0.05 ^d^	2.60 ± 0.01 ^d^	6.11 ± 0.02 ^b^
Est. CATs	27.12 ^b^	28.68 ^b^	6.51 ^c^	4.18 ^d^	33.80 ^a^
Non-est. CATs	9.61 ^c^	11.28 ^b^	4.32 ^d^	2.49 ^e^	13.68 ^a^
ECDs	33.14 ^b^	34.70 ^b^	8.61 ^c^	5.36 ^d^	42.99 ^a^
Total Catechins	37.26 ^bc^	41.33 ^b^	11.25 ^d^	7.21 ^d^	48.46 ^a^
CAF	22.76 ± 0.33 ^c^	16.66 ± 1.24 ^d^	22.25 ± 1.19 ^c^	29.75 ± 2.17 ^b^	55.22 ± 6.08 ^a^
Theaflavins					
TF	8.32 ± 0.13 ^a^	6.21 ± 0.11 ^c^	6.91 ± 0.06 ^b^	6.13 ± 0.10 ^c^	6.96 ± 0.13 ^b^
TF-3-G	9.92 ± 0.06 ^a^	7.64 ± 0.37 ^b^	7.97 ± 0.04 ^b^	6.60 ± 0.23 ^c^	7.99 ± 0.23 ^b^
TF-3’-G	9.73 ± 0.08 ^a^	7.81 ± 0.11 ^c^	7.26 ± 0.04 ^c^	6.06 ± 0.11 ^d^	8.89 ± 0.23 ^b^
TF-3,3’-DG	8.76 ± 0.17 ^b^	6.53 ± 0.20 ^c^	6.20 ± 0.10 ^c^	5.58 ± 0.19 ^d^	9.04 ± 0.10 ^a^
Total TFs	36.73 ± 0.77 ^a^	28.18 ± 0.80 ^c^	28.33 ± 0.73 ^c^	24.37 ± 0.42 ^d^	32.87 ± 0.96 ^b^

^1^ Values are mean ± SD (*n* = 3). Different superscript letters indicate that values are significantly different (*p* < 0.05) between zones; GC, gallocatechin; EGC, epigallocatechin; C, catechin; EC, epicatechin; EGCG, epigallocatechin-3-gallate; GCG, gallocatechin-3-gallate; ECG, epicatechin-3-gallate; GA, gallic acid; CAF, caffeine; Est. CATs (esterified catechins), sum of EGCG, ECG, and GCG; Non-est. CATs (non-esterified catechins), sum of C, EC, EGC, and GC; ECDs (epicatechin derivatives), sum of EC, EGC, EGCG, and ECG. Total Catechins, sum of GC, EGC, C, EC, EGCG, GCG, ECG; TF, theaflavin; TF-3-G, theaflavin-3-*O*-gallate; TF-3’-G, theaflavin-3’-*O*-gallate; TF-3,3′-DG, theaflavin-3,3′-*O*-digallate; TFs, sum of TF, TF-3-G, TF-3’-G, and TF-3,3′-DG; DE, dry extract; DW, dry weight.

## Data Availability

All available data are reported in this paper.

## Supplementary Materials

The following supporting information can be downloaded at: https://www.mdpi.com/article/10.3390/plants14010103/s1, Table S1: Analytical parameters of the developed HPLC method for EGC, EGCG and ECG. Table S2: Analytical parameters of the developed HPLC method for theaflavins.
