# Supplementary material for: Phytochemicals and Biological Properties of Azorean Camellia sinensis Black Tea Samples from Different Zones of Tea Plantation
Source: Plants (Basel). 2025 Jan 2;14(1):103. doi: 10.3390/plants14010103 (PMC11723068; doi:10.3390/plants14010103)
Supplement: Supplementary file 1 [file plants-14-00103-s001.zip › plants-3381950-supplementary.pdf]

Table S1. Analytical parameters of the developed HPLC method for EGC, EGCG and ECG.

ECDs	Linear Range (mg/L)	R^2	LOD (mg/L)	LOQ (mg/L)	Recovery (%)	Inter-day/Intra-day (% RSD)
EGC	0.038–0.613	0.9965	0.02–0.15	0.05–0.40	96.2–102.4	2.5/0.95
EGCG	0.143–2.292	0.9995	0.01–0.06	0.05–0.25	98.0–99.8	3.10/1.15
ECG	0.041–1.327	0.9982	0.02–0.20	0.06–0.55	95.3–103.2	3.80/1.90

R^2 –correlation coefficient; LOD–limit of detection (inj. vol. = 5 μL); LOQ–limit of quantification (inj. vol. = 5 μL).

Table S2. Analytical parameters of the developed HPLC method for theaflavins.

ECDs	Linear Range (mg/L)	R^2	LOD (mg/L)	LOQ (mg/L)	Recovery (%)	Inter-day/Intra-day (% RSD)
TF	0.24–95.00	0.9995	0.18–0.26	0.69–0.97	96.1–99.3	0.63/0.02
TF-3-G	0.32–96.4	0.9994	0.16–0.22	0.52–0.74	97.2–100.1	0.48/0.06
TF-3′-G	0.22–95.55	0.9993	0.25–0.35	0.89–1.25	97.3–99.7	0.53/0.07
TF-3,3′-DG	0.20–97.00	0.9999	0.10–0.15	0.33–0.47	95.6–100.2	0.63/0.07

R^2 –correlation coefficient; LOD–limit of detection (inj. vol. = 12.5 μL); LOQ–limit of quantification (inj. vol. = 12.5 μL).
